# Arsenical keratoses with Bowen disease in Kuwait revealing transnational household well-water exposure

**DOI:** 10.1016/j.jdcr.2026.07.062

**Published:** 2026-08-07

**Authors:** Hashem Boalbanat, Ahmad Alsarraf, Daniah Hassan, Mohammad Hasan

**Affiliations:** Ministry of Health, Kuwait Institute for Medical Specialization, Kuwait City, Kuwait

**Keywords:** arsenical keratosis, Bangladesh, Bowen disease, chronic arsenicosis, migrant health, raindrop pigmentation, squamous cell carcinoma in situ, well-water arsenic

## Introduction

Arsenical keratoses are precancerous skin findings of chronic arsenic toxicity. They classically present as yellow-brown punctate palmoplantar hyperkeratoses with diffuse hyperpigmentation and guttate hypopigmented macules, producing a “raindrop on a dusty road” appearance. Bowen disease, squamous cell carcinoma, and basal cell carcinoma may occur in chronically exposed patients.^1^^,^^2^ Bangladesh has one of the largest documented population exposures to arsenic contaminated well-water.^3^ In nonendemic settings, including Gulf States such as Kuwait, chronic arsenicosis may be encountered as an imported dermatosis, and diagnosis may be difficult when exposure ended years before presentation.^4^ We describe a Bangladeshi construction worker in Kuwait whose classic skin findings, biopsy-proven Bowen disease, and shared household exposure supported chronic arsenicosis.

## Case report

A 41-year-old Bangladeshi construction worker living in Kuwait for approximately 20 years presented with 3 decades of progressive generalized dyspigmentation and palmoplantar hyperkeratosis. Skin darkening began in adolescence in his home village, followed by guttate hypopigmented macules, palmoplantar thickening, and several enlarging dark plaques on the trunk over the preceding 1 to 2 years. He denied constitutional symptoms, mucosal symptoms, and nail changes.

**Fig 1:**
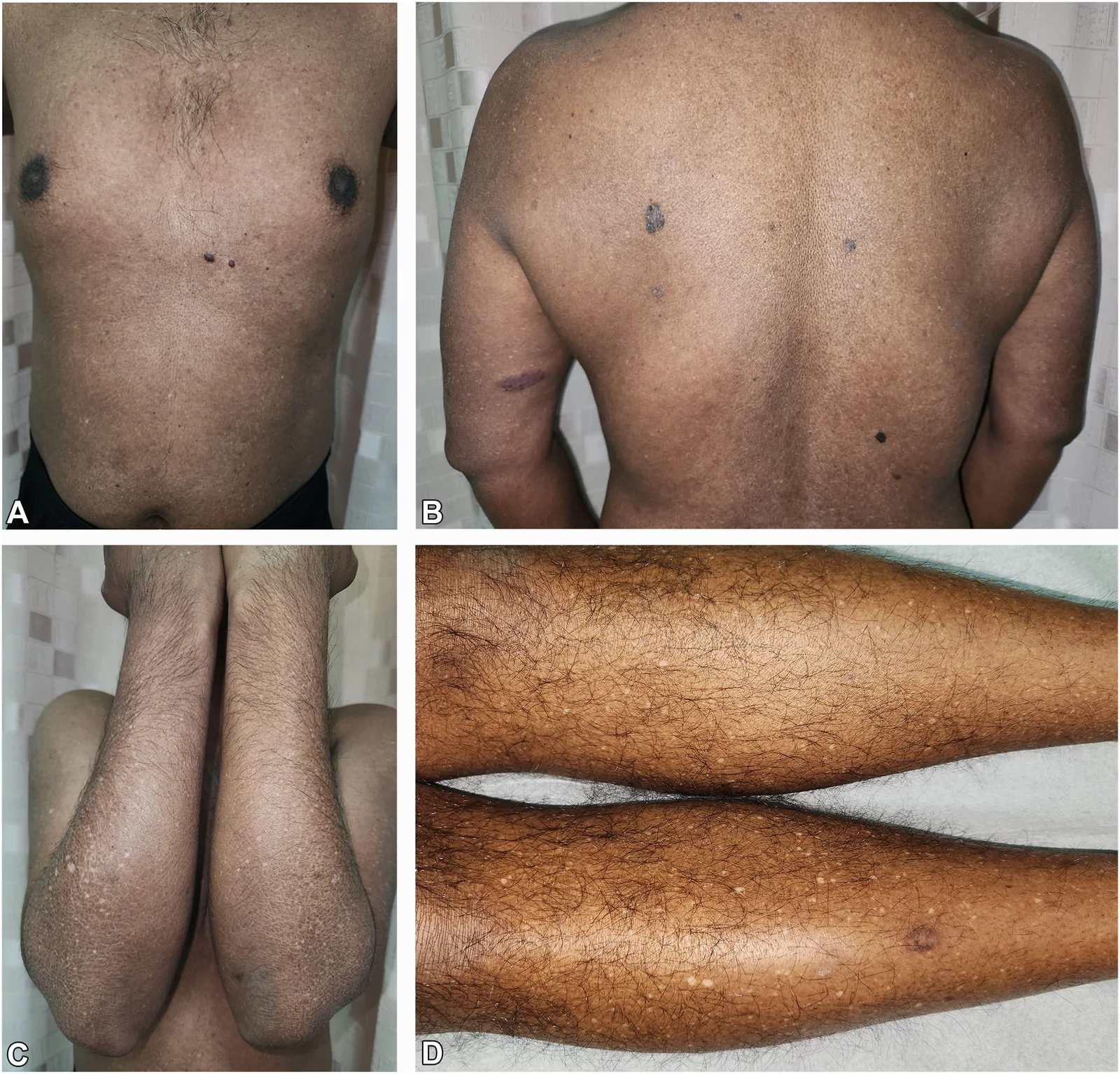
Arsenical dyschromia. **A,** Anterior trunk showing diffuse brown hyperpigmentation studded with innumerable symmetrically distributed 1- to 4-mm guttate hypopigmented macules, the classic raindrop-on-a-dusty-road pattern. **B,** Back showing similar diffuse truncal dyschromia with several superimposed brown-black hyperkeratotic plaques (representative biopsy site visible on the left scapular region; see Fig 3, *B* for close-up). **C,** Bilateral extensor elbows showing xerotic hyperkeratotic roughening with scattered guttate hypopigmented macules. **D,** Lower legs showing innumerable hypopigmented macules on a hyperpigmented background.

**Fig 2:**
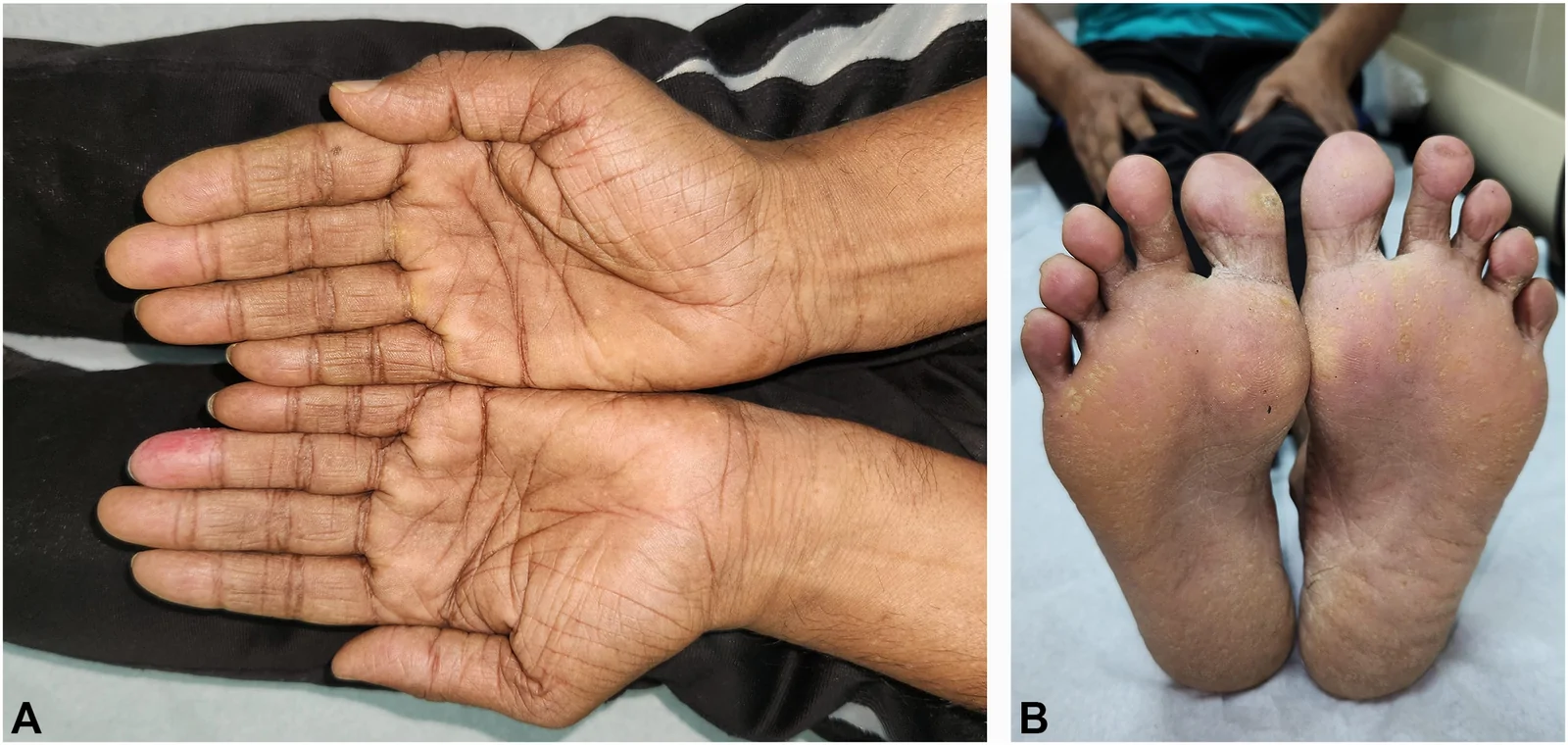
Palmoplantar arsenical keratoses. **A,** Bilateral palms showing diffuse keratoderma with accentuated dermatoglyphics and subtle yellow punctate keratotic papules along the volar fingers. **B,** Bilateral soles showing diffuse plantar keratoderma with multiple discrete yellow hyperkeratotic papules and plaques concentrated over pressure-bearing skin of the toes, metatarsal heads, and medial heels.

**Fig 3:**
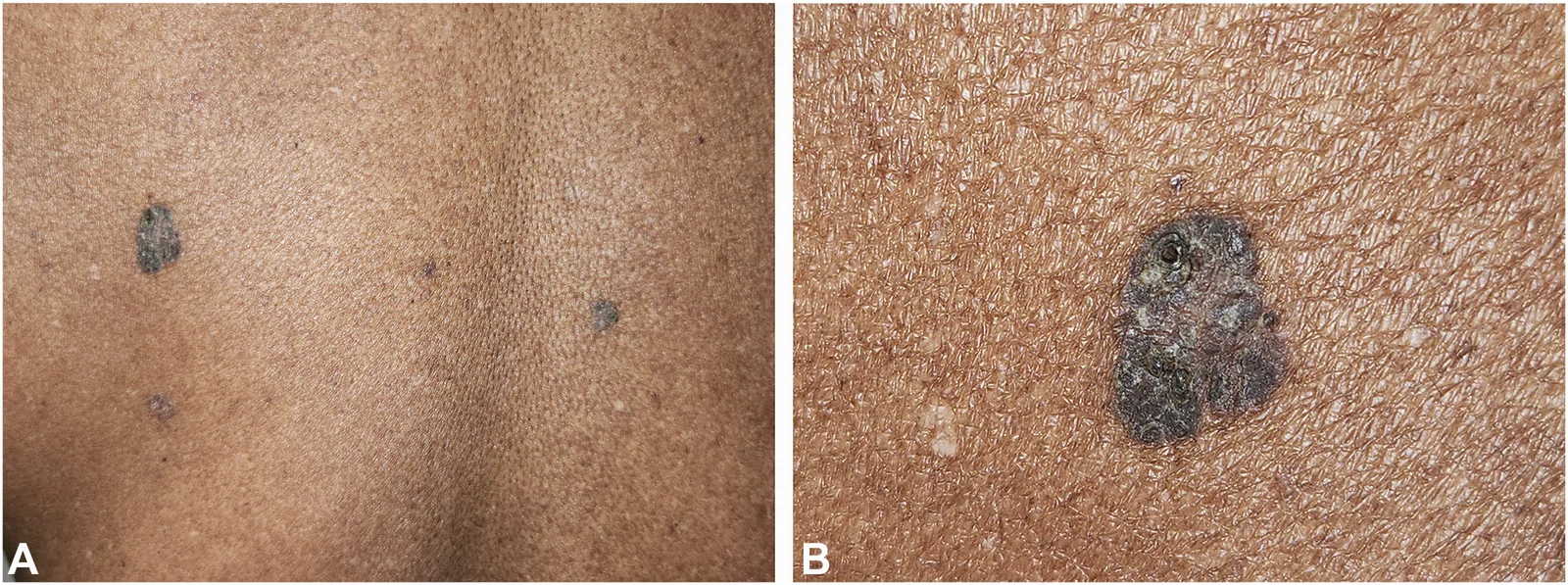
Arsenic associated truncal hyperkeratotic plaques with biopsy proven Bowen disease. **A,** Mid back showing several brown black hyperkeratotic plaques on a background of arsenical dyschromia. **B,** Close up of the biopsied truncal plaque showing an irregular hyperpigmented verrucous surface with adherent crust; histopathology confirmed squamous cell carcinoma in situ.

Examination showed diffuse brown hyperpigmentation with innumerable sharply demarcated 1 to 4 mm hypopigmented macules over the trunk and extremities, producing the classic “raindrop on a dusty road” pattern (Fig 1, *A* and *B*). Xerotic hyperkeratotic change was present on the extensor elbows, and similar color change involved the lower legs (Fig 1, *C* and *D*). The palms showed diffuse keratoderma with accentuated dermatoglyphics and subtle yellow punctate keratotic papules. The soles showed multiple yellow hyperkeratotic papules concentrated over weight-bearing skin (Fig 2). Several brown-black hyperkeratotic plaques were present on the upper back and chest (Fig 3, *A*). One plaque on the left upper back, visible in Figs 1, *B* and 3, *A*, had an irregular hyperpigmented verrucous surface with adherent crust on close-up view and was biopsied because of concern for squamous cell carcinoma in situ (Fig 3, *B*). Mucosal, nail, and systemic examinations were unremarkable.

**Fig 4:**
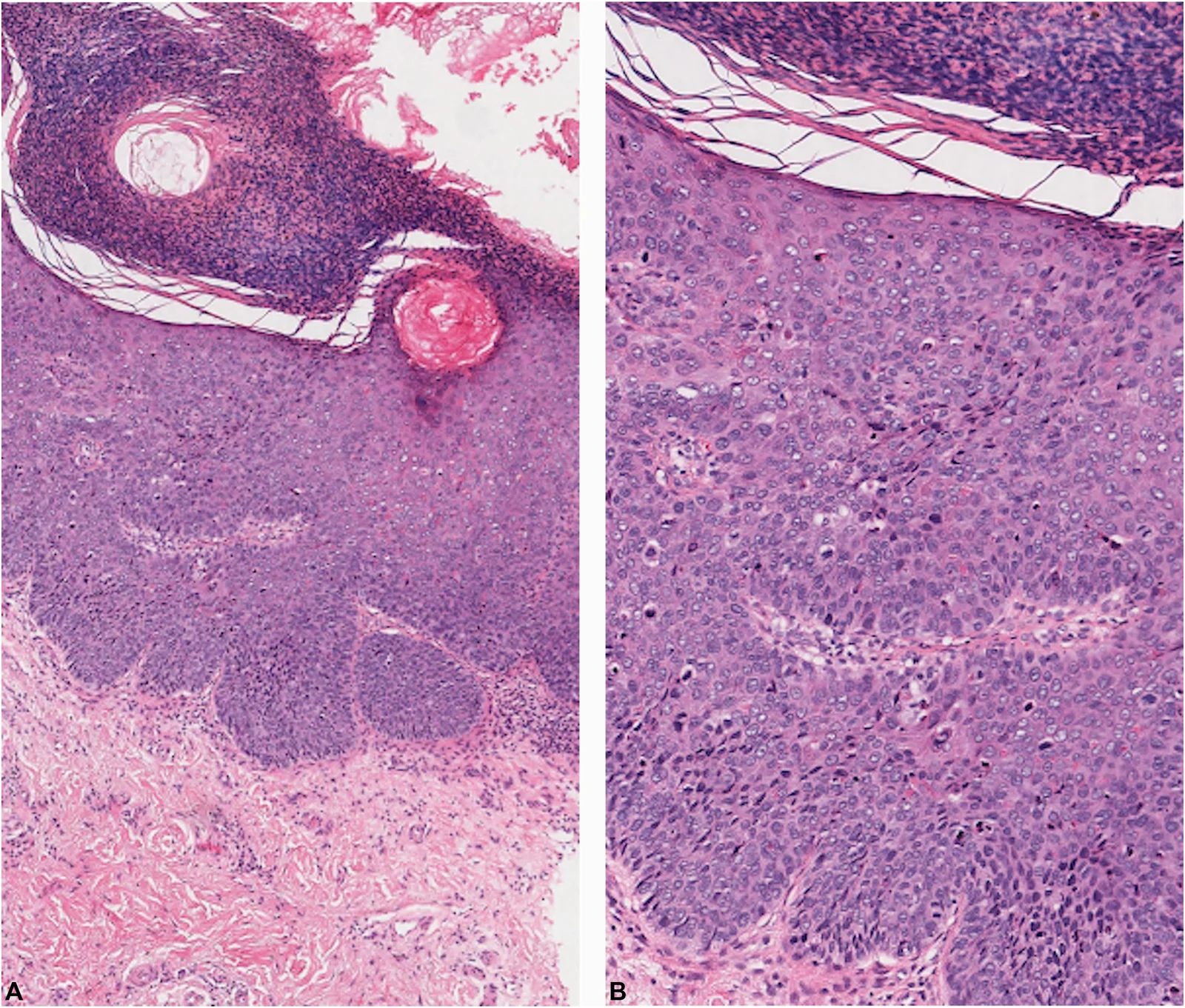
Histopathology of a hyperkeratotic pigmented truncal plaque (hematoxylin-eosin). **A,** Lower-power view, original magnification × 4 showing compact hyperkeratosis with focal parakeratotic scale-crust, acanthosis, and full-thickness epidermal atypia with an intact dermoepidermal junction and no invasive component. **B,** Higher-power view, original magnification × 10 view showing disorderly full-thickness atypia with loss of maturation and polarity, enlarged pleomorphic hyperchromatic keratinocytes, scattered dyskeratotic and apoptotic keratinocytes**,** and mitotic figures, consistent with squamous cell carcinoma in situ (Bowen disease).

Routine laboratory studies and chest radiography were within reference ranges. Punch biopsy of the indurated truncal plaque showed compact hyperkeratosis with focal parakeratotic scale, acanthosis, and full thickness epidermal atypia with loss of maturation and polarity, pleomorphic hyperchromatic keratinocytes, scattered dyskeratosis, and mitotic figures. The dermoepidermal junction was intact, and no invasive component was identified. These findings were consistent with squamous cell carcinoma in situ (Bowen disease) (Fig 4).

Blood, urine, hair, and nail arsenic assays were unavailable at our center. Directed exposure history was therefore critical. The patient denied arsenic containing traditional remedies and occupational arsenic exposure in Kuwait. He reported that several relatives and neighbors from his home village had similar pigmentary and keratotic changes after lifelong consumption of water from the same village well. He also reported that an older paternal uncle from the same household, now residing in the United Kingdom, had similar skin findings and had been told during medical evaluation that his arsenic level was elevated. This coexposed household history supported a shared environmental exposure, although confirmatory testing could not be performed in the index patient. The combination of classic clinical morphology, biopsy-proven Bowen disease, shared household well exposure, and arsenic testing in a coexposed relative supported a diagnosis of chronic arsenicosis with arsenical keratoses and Bowen disease.

Acitretin 25 mg daily was started for extensive keratotic disease. The biopsy proven Bowen disease was treated with cryotherapy. The patient was counseled regarding photoprotection, long-term dermatologic surveillance, and evaluation of new or changing lesions. Treatment response and malignancy/neurological surveillance were interrupted by repatriation; records were provided to continue care abroad, with advice to encourage screening and contact tracing of potentially coexposed household members.

## Discussion

The value of this case lies in the diagnostic route. A classic clinicopathologic presentation in a nonendemic Gulf practice setting prompted reconstruction of remote village exposure. The history was further supported by the report of a similarly affected coexposed relative who had migrated to a third country. This family link changed an apparently isolated dermatologic presentation in Kuwait into evidence of a shared household environmental exposure from well-water consumption.

The bedside morphology was highly characteristic. The combination of raindrop dyspigmentation and palmoplantar punctate keratoses is highly suggestive of chronic arsenicosis.^1^^,^^5^ Punctate palmoplantar keratoderma, verruca vulgaris, and other acquired keratodermas may mimic the keratotic lesions but would not explain the concurrent guttate hypopigmentation on a hyperpigmented background. Biopsy remains essential for suspicious plaques because Bowen disease, squamous cell carcinoma, and basal cell carcinoma may resemble seborrheic keratosis or other verrucous neoplasms.^1^^,^^2^ In Tao and Wang’s review, 52.6% of reported patients with arsenical keratosis had associated cutaneous tumors.^2^

A second practical point is the limited value of toxicologic testing when exposure is remote. Blood and urinary arsenic reflect recent ingestion, whereas hair and nail arsenic generally reflect exposure over the preceding months.^6^, ^7^, ^8^ A patient who left the source region more than a decade earlier may therefore have nondiagnostic biological testing despite a highly characteristic phenotype.^6^^,^^7^ A limitation is that arsenic testing in the coexposed relative was reported by the patient and could not be independently verified. In remote exposure settings, targeted questioning about coexposed relatives may still provide useful support, particularly when relatives have undergone arsenic testing elsewhere. Other recognized exposure pathways include arsenic-adulterated traditional Chinese medicine, indoor coal combustion and arsenic based pesticides.^2^^,^^9^^,^^10^

Management priorities include removal of any ongoing exposure, treatment of symptomatic keratoses, treatment of established neoplasms, and long-term surveillance for additional cutaneous malignancies.^1^^,^^7^ Systemic retinoids such as acitretin have been reported to improve arsenical keratoses and may be considered for extensive keratotic disease or field cancerization.^2^

For dermatologists in the Gulf and other regions that care for migrants, the practical message is simple: palmoplantar punctate keratoses with raindrop dyspigmentation should prompt targeted questioning about remote residence in arsenic-endemic regions, well-water source, and similar skin findings or arsenic testing in coexposed relatives. Recognition of this pattern can uncover arsenic exposure long after the patient has left the source.

### Declaration of generative AI and AI-assisted technologies in the writing process

During the preparation of this work the authors used a generative AI language tool to assist with language refinement and organization of the manuscript draft. After using this tool, the authors reviewed and edited the content as needed and take full responsibility for the content of the published article.

## Conflicts of interest

None disclosed.

## References

[1] Shajil C., Chen R.J., Mahabal G.D. (2024). *StatPearls* [Internet].

[2] Tao R., Wang R. (2024). Arsenical keratosis in China: a case report and review of the literature. Skin Res Technol.

[3] Smith A.H., Lingas E.O., Rahman M. (2000). Contamination of drinking-water by arsenic in Bangladesh: a public health emergency. Bull World Health Organ.

[4] Kaur T., Budhwar J. (2021). Arsenical keratoses: case report from nonendemic area of Amritsar. Clin Dermatol Rev.

[5] Sengupta S.R., Das N.K., Datta P.K. (2008). Pathogenesis, clinical features and pathology of chronic arsenicosis. Indian J Dermatol Venereol Leprol.

[6] Das N.K., Sengupta S.R. (2008). Arsenicosis: diagnosis and treatment. Indian J Dermatol Venereol Leprol.

[7] Tang G.T., Elakis J., Scardamaglia L. (2023). Cutaneous manifestations and treatment of arsenic toxicity: a systematic review. Skin Health Dis.

[8] Adair B.M., Hudgens E.E., Schmitt M.T., Calderon R.L., Thomas D.J. (2005). Total arsenic concentrations in toenails quantified by two techniques provide a useful biomarker of chronic arsenic exposure in drinking water. Environ Res.

[9] Yao M., Zeng Q., Luo P. (2021). Assessing the risk of coal-burning arsenic-induced liver damage: a population-based study on hair arsenic and cumulative arsenic. Environ Sci Pollut Res Int.

[10] Li Y., Ye F., Wang A. (2016). Chronic arsenic poisoning probably caused by arsenic-based pesticides: findings from an investigation study of a household. Int J Environ Res Public Health.

